# An Energy Efficient UAV-Based Edge Computing System with Reliability Guarantee for Mobile Ground Nodes

**DOI:** 10.3390/s21248264

**Published:** 2021-12-10

**Authors:** Seung-Yeon Kim, Yi-Kang Kim

**Affiliations:** Department of Computer Convergence Software, Korea University, Sejong 30019, Korea; kimsy8011@korea.ac.kr

**Keywords:** unmanned aerial vehicle (UAV), edge computing, mobile ground node (MGN), energy efficiency, reliability

## Abstract

An edge computing system is a distributed computing framework that provides execution resources such as computation and storage for applications involving networking close to the end nodes. An unmanned aerial vehicle (UAV)-aided edge computing system can provide a flexible configuration for mobile ground nodes (MGN). However, edge computing systems still require higher guaranteed reliability for computational task completion and more efficient energy management before their widespread usage. To solve these problems, we propose an energy efficient UAV-based edge computing system with energy harvesting capability. In this system, the MGN makes requests for computing service from multiple UAVs, and geographically proximate UAVs determine whether or not to conduct the data processing in a distributed manner. To minimize the energy consumption of UAVs while maintaining a guaranteed level of reliability for task completion, we propose a stochastic game model with constraints for our proposed system. We apply a best response algorithm to obtain a multi-policy constrained Nash equilibrium. The results show that our system can achieve an improved life cycle compared to the individual computing scheme while maintaining a sufficient successful complete computation probability.

## 1. Introduction

### 1.1. Motivation

For effective data computation, cloud and edge computing have been used in a rapidly growing variety of applications. In traditional centralized computing system architectures, data are backhauled to and processed in either a central enterprise data center or the cloud [1]. By contrast, in a distributed computing framework, an edge computing system provides efficient data computation at the edge of the network. This ensures that processing occurs in real-time without the need for the cloud or external data centers, as vehicles such as trains, planes, and connected cars can support the edge computing service [2].

Traditional infrastructure-based mobile edge computing (MEC) is a technology widely used in resource-constrained applications such as IoT. However, the unchangeable and limited wireless coverage of infrastructure-based MEC severely hampers service flexibility and efficiency [3]. On the other hand, an unmanned aerial vehicle (UAV)-aided edge computing system provides a way to solve the above problem. UAV-aided edge computing, which is characterized by large area coverage, line-of-sight communication, and customizable cruising, provides flexible and low-cost edge services for a large number of widely deployed IoT devices on the ground [4]. For UAV-aided edge computing, UAV-aided architectures have been proposed [5,6,7], where a UAV serves as a node that is involved in various tasks—for instance, an edge computing server that executes the computational tasks of nodes, and a relay station to offload computational tasks [8]. Although UAVs provide some advantages to edge computing systems, such systems still need guaranteed reliability to enhance the computational task completion and efficient energy management of the system [9,10]. As the energy of a UAV is typically limited, there may be instances when the battery capacity level of the UAV is not sufficient for computation, and the UAV may therefore provide an incomplete computation service to the user. In addition, for data transmission from the node to the UAV or from the UAV to the node, transmission failures such as packet loss and bit error can occur as a result of the stochasticity of the wireless channels or the movement of the user [11,12].

Our aim was to design an energy efficient edge computing system with guaranteed reliability for computational task completion. Consider an autonomous vehicle system involving safety production monitoring, automatic driving, and a cooperative-intelligent transport system in a smart city [13,14,15]. To operate such a system, the mobile ground node (MGN) should collect and compute data from multiple sources (traffic signals, roadside stations, autonomous vehicles, etc.). Then, because most MGNs have limited computing capabilities, they can offload their computational tasks to a UAV acting as an edge server to improve the computing efficiency, because the data require high performance computing, such as high-resolution image processing, video processing, pattern recognition, stream data mining, and online task planning. However, even if the MGN requests a computing service from a UAV, as mentioned above, there are still issues with the reliability of computational task completion and the energy management of such a system.

### 1.2. Related Work and Main Contribution

Various studies have attempted to improve the energy efficiency and processing reliability of edge computing systems [16,17,18,19,20,21,22,23,24]. For example, reference [16,17,18] introduced optimization problems for an energy-efficient computation offloading in UAV-based edge computing systems. In [16], to minimize the energy consumption of the UAV’s data processing, the computational offloading problem was formulated with a time-duration constraint for CPU cycles. Meanwhile, Li et al. [17] developed a model to maximize the energy efficiency of a UAV by jointly optimizing the UAV trajectory, user transmit power, and computation load allocation. In another study, You et al. [18] designed an energy-efficient offloading policy for determining the offloading data volume, offloading duration, and transmission resources of each user. Further, Hua et al. [19] proposed a collaborative scheduling scheme that is jointly optimized by bit allocation, resource partitioning, and power allocation at the user devices/UAV to minimize the total energy consumption of user devices. To increase the reliability of UAV-based communication, reference [20,21,22,23,24] investigated the wireless channel and mobility. Al-Hourani et al. [20] established a link channel probability model—which makes mobility determinations based on the angle from the ground—and proposed an optimal UAV deployment that maximizes the coverage area. The path loss and channel gain of the link between a UAV and a ground node were examined in [21]. In the context of link channel probability, [22,23] studied secrecy capacity UAV-aided communication systems and UAV-to-ground communication in the presence of an interferer, respectively. Further, Kim et al. [24] established a foundation for multi-layer aerial networks where each layer has UAVs with different densities, floating altitudes, and transmission powers. However, no previous work has optimized the energy efficiency and processing reliability of edge computing systems in a distributed manner. Additionally, reference [25,26,27] considered the UAV-based edge computing system to achieve the minimum energy consumption and delay. In [25], the authors designed a collaborative UAV network system, which helps isolated vehicle routing and load balancing by using a prediction of vehicle distributions. In [26], the authors considered the pareto optimal solution that balances the trade-off between the completion time and energy consumption of a UAV. In [27], the authors proposed multi-user offloading for edge computing networks, in which users select the best edge server to offload their tasks to achieve the minimum delay.

In this paper, we propose a UAV-based edge computing system with an energy harvesting capability [28,29]. In our proposed system, when the MGNs need to compute the data, they send them to geographically proximate multiple UAVs, because this system uses the additional assistance of UAVs to improve the reliability for computational task completion. However, the performance of excessively redundant computing processes by the UAVs can decrease their energy efficiency in the system. Therefore, in our scheme, when the MGN sends a computational task service request to the UAVs, the UAVs determine whether or not to compute the MGN’s data in a distributed manner. As one UAV cannot know the decisions made by the other UAVs, some degree of redundant computing may occur. Accordingly, to optimize the trade-off between reliability for computational task completion and energy efficiency, a stochastic game model with constraints was formulated, and a multi-policy constrained Nash equilibrium is obtained by a best response algorithm. The results show that, compared to the individual computing scheme, our system can achieve an improved life cycle while maintaining a sufficiently complete computation service. Although the authors in [30] formulated a constrained stochastic game model [31] for an energy-efficient Internet of Things system, their model only considers the energy consumption and error of one-way data transmission.

The three key contributions of our paper can be summarized as follows.

1. We studied our energy efficient edge computing system which has a reliability guarantee via multiple UAVs located near an MGN, selectively executing computational tasks. Unlike the conventional centralized approaches, for our proposed system, we formulated a stochastic game model with constraints to obtain the optimal policy regarding the behavior of UAVs in a distributed manner.
2. In contrast to [27,30], our scheme considers the energy consumption of the UAV, the desired probability of completing the processing according to the bidirectional data transmission error, and the number of cooperative UAVs in the edge computing system.
3. Simulation results are provided to validate the performance of the proposed designs, and illustrate the energy consumption of the UAV and complete computation probability. Besides, the simulation results show that the proposed design provides a significant improvement in terms of cluster lifetime for the considered UAV-aided system compared to the baseline schemes.

The rest of the paper is organized as follows. Section 2 describes the UAV-based edge computing system. In Section 3, the constrained stochastic game model is formulated. Section 4 presents numerical examples, and concluding remarks are provided in Section 5.

## 2. System Model

**Figure 1:**
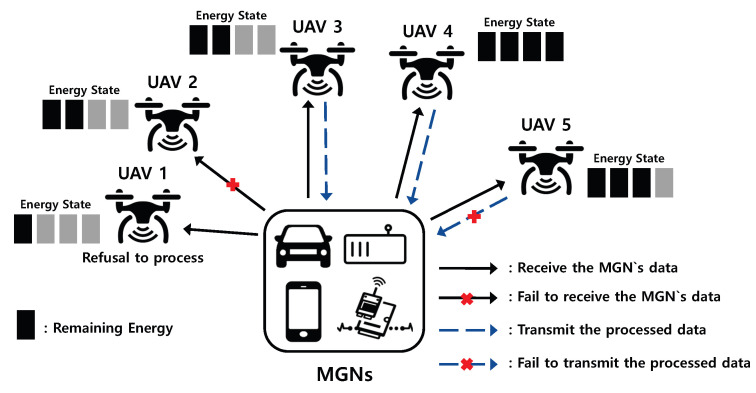
Example of a UAV-based edge computing system scenario.

Figure 1 shows an example of a UAV-based edge computing system scenario wherein each UAV can execute the particular computational task required of it for high performance computing; this system also has energy harvesting capabilities. In addition, we assume that one unit of energy of the UAV is consumed when receiving the MGN’s data, and another is consumed when processing the data and then sending them. In general, the transmission power and receiving power of a node can be calculated by multiplying the transmission power of the current node by the transmission time. Meanwhile, the transmission time can be obtained from the total volume of data [32]. Our system is based on federated learning concept, where UAVs perform the model training for a raw dataset received from the MGN. After training, each UAV transfers its local model parameters to the MGN; these model parameters constitute a small amount of data. In our system, since the volume of raw dataset is the same as that of the processed, we assume that the sending and task processing power consumes one unit.

As shown in Figure 1, the MGN transmits the computational task to multiple UAVs; since UAV 1 has the minimum remaining energy state, it refuses to receive the MGN’s data, and UAVs with sufficient energy thus receive the task of the MGN. However, in this receiving process, as a result of a receiving error, UAV 2 cannot execute the task, though one unit of power is still consumed. For UAV 5, after receiving and computing the MGN’s data, the computing service remains uncompleted as a result of a sending error, although two units of power are still consumed. For UAV 3 and UAV 4, a sufficiently complete computation service is achieved by which two units of power are consumed. After receiving the processed data from UAVs, the MGN seeks a consensus on the processed data using an average algorithm, such as the FedAvg algorithm for federated learning, which is a well-established method for performing machine learning tasks over distributed data [33]. For example, when the MGN receives *M* model parameters from the UAVs that participated in the data computing, it can obtain the result, *R*, as follows:

(1) $$\begin{array}{c} R=\frac{1}{M}\sum_{m=1}^{M}\omega_{m}, \end{array}$$

where $\omega_{m}$ is a model parameter received by UAV *m*.

In our system, to alleviate the degradation of the successful complete computation probability caused by transmission error, several UAVs can redundantly process the MGN’s data, even though the MGN only needs one set of processed data; these excessively redundant data computations and transmissions reduce the energy efficiency of UAVs. Therefore, in our proposed system, each UAV should consider the actions of other UAVs in determining whether or not to execute the MGN’s computation tasks.

## 3. Game Model and Optimization Formulation

In a stochastic game model with constraints, UAVs and players are used interchangeably. For *N* UAVs, each UAV can be defined by the tuple $\left\{S^{i},A^{i},P^{i},v,c\right\}$ for *i*, where i∈{1,…,N}.

- $S^{i}$ is a finite local state space of UAV *i*, where $S^{i}=\left\{0,1,\ldots ,E_{max}\right\}$ for the maximum energy capacity of UAVs $E_{max}$. Then, $S=\prod_{i}S^{i}$ is a global state space, where ∏ is the Cartesian product. In addition, $S^{-i}=\prod_{j\neq i}S^{j}$ is the state space of all UAVs excluding UAV *i*.
- $A^{i}$ is a finite local action set of UAV *i*, where $A^{i}\in \left\{0,1,2\right\}$ represents refusing to compute, receiving the MGN’s data, and transmitting the processed data after computing, respectively.
- $P^{i}$ is the transition probability of UAV *i*, where $P^{i}\left[S^{i^{\prime }}|S^{i},A^{i}\right]$ is the probability that the state of UAV *i* moves from state $S^{i}$ to $S^{i^{\prime }}$ if it chooses an action $A^{i}$.
- ν is the cost function defined to minimize the energy consumption. As mentioned previously, since the UAV consumes one unit of energy to receive the MGN’s data, and since it consumes another unit of energy to process the data and then transmit the processed data, $\nu (S^{i},A^{i})=1$ when $A^{i}=$1 or 2 while $\nu (S^{i},A^{i})=0$ for $A^{i}=0$.
- *c* is the constraint function to represent the successful complete computation probability while accounting for transmission errors. The computation of the MGN’s data can be successfully completed if there is at least one successful complete computation between a UAV and the MGN. Therefore, if the target UAV *i* chooses $A^{i}=2$ and has $S^{i}>1$, then the successful complete computation probability can be represented as
  (2) $$\begin{array}{c} \left\{\begin{array}{cc} \left(1-\epsilon_{Rx}\right)\left(1-\epsilon_{Tx}\right)+\left(1-\epsilon_{Rx}\right)\epsilon_{Tx}\left(1-\epsilon_{Rx}\right)\left(1-\epsilon_{Tx}\right)\lambda^{-i} & \\ \;\;\;\;\;\;\;\;\;\;\;\;\;\;\;\;\;\;\;\;\;\;\;\;\;\;\;\;\;\;\;\;\;\;\;+\epsilon_{Rx}\left(1-\epsilon_{Rx}\right)\left(1-\epsilon_{Tx}\right)\lambda^{-i}, & \mathrm{if}\;S^{i}>1,A^{i}=2\\ \left(1-\epsilon_{Rx}\right)\left(1-\epsilon_{Tx}\right)\lambda^{-i}, & \mathrm{otherwise}, \end{array}\right. \end{array}$$
  where $\lambda^{-i}$ denotes the probability that at least one UAV aside from UAV *i* receives the MGN’s data. In addition, $\epsilon_{Rx}$ and $\epsilon_{Tx}$ denote the respective probabilities of suffering a receiving error or a transmitting error (We assume that the channels between UAVs and nodes are the same quality. In UAV-aided computing system, UAVs can fly close to mobile ground nodes. When UAVs fly close enough with better channel quality, it is possible that UAVs or nodes can efficiently receive/transmit data and have same channel quality. However, transmission failures such as packet loss and bit error can occur from the stochasticity of the wireless channels or the mobility of the user.).

### 3.1. Transition Probability

The transition probability of the target UAV *i* introduced above can be derived as follows.

When UAV *i* does not execute the MGN’s computation task (i.e., $A^{i}=0$ ) and the energy of UAV *i* is not fully charged (i.e., $S^{i}\neq E_{max}$), its energy increases by one unit. The UAV can harvest energy only when its environment provides energy. Therefore, the energy harvested by the UAV can be modeled by a Bernoulli random process taking values in {0,1}, and the probability that the UAV harvests one unit energy is $P_{H}$. When UAV *i* does not execute the MGN’s computation task (i.e., $A^{i}=0$ ) and the energy of the UAV is fully charged (i.e., $S^{i}=E_{max}$), it no longer harvests energy. Therefore, the corresponding probabilities can be expressed by

(3) $$\begin{array}{c} P^{i}\left[S^{i^{\prime }}|S^{i}\neq E_{max},A^{i}=0\right]=\left\{\begin{array}{cc} P_{H}, & \mathrm{if}\;S^{i^{\prime }}=S^{i}+1\\ 1-P_{H}, & \mathrm{if}\;S^{i^{\prime }}=S^{i}\\ 0, & \mathrm{otherwise} \end{array}\right. \end{array}$$

and

(4) $$\begin{array}{c} P^{i}\left[S^{i^{\prime }}|S^{i}=E_{max},A^{i}=0\right]=\left\{\begin{array}{cc} 1, & \mathrm{if}\;S^{i^{\prime }}=S^{i}\\ 0, & \mathrm{otherwise}. \end{array}\right. \end{array}$$

When UAV *i* receives the MGN’s data and it has two or more units of energy, it consumes one unit of energy. On the other hand, if UAV *i* has fewer than two energy units, it cannot execute the MGN’s computation task, and it thus consumes no energy. In addition, its energy increases by one unit with $P_{H}$. Therefore, the corresponding probabilities can be represented as

(5) $$\begin{array}{c} P^{i}\left[S^{i^{\prime }}|S^{i}>1,A^{i}=1\right]=\left\{\begin{array}{cc} P_{H}, & \mathrm{if}\;S^{i^{\prime }}=S^{i}\\ 1-P_{H}, & \mathrm{if}\;S^{i^{\prime }}=S^{i}-1\\ 0, & \mathrm{otherwise} \end{array}\right. \end{array}$$

and

(6) $$\begin{array}{c} P^{i}\left[S^{i^{\prime }}|S^{i}\leq 1,A^{i}=1\right]=\left\{\begin{array}{cc} P_{H}, & \mathrm{if}\;S^{i^{\prime }}=S^{i}+1\\ 1-P_{H}, & \mathrm{if}\;S^{i^{\prime }}=S^{i}\\ 0, & \mathrm{otherwise}. \end{array}\right. \end{array}$$

When UAV *i* receives the MGN’s data without a receiving error while having sufficient energy, UAV *i* can execute the task and transmit the processed data to the MGN, and one unit of energy is consumed. On the other hand, if UAV *i* receives the data with an error or does not have sufficient energy, it cannot process the data, and it thus consumes no energy. Therefore, the corresponding probabilities can be represented as

(7) $$\begin{array}{c} P^{i}\left[S^{i^{\prime }}|S^{i}\neq 0,A^{i}=2\right]=\left\{\begin{array}{cc} 1, & \mathrm{if}\;S^{i^{\prime }}=S^{i}-1\\ 0, & \mathrm{otherwise}. \end{array}\right. \end{array}$$

and

(8) $$\begin{array}{c} P^{i}\left[S^{i^{\prime }}|S^{i}=0,A^{i}=2\right]=\left\{\begin{array}{cc} 1, & \mathrm{if}\;S^{i^{\prime }}=S^{i}\\ 0, & \mathrm{otherwise}. \end{array}\right. \end{array}$$

### 3.2. Optimization Formulation

The stationary multi-policy of all UAV, π, is to be optimized such that the constraints of long-term average energy consumption and long-term average successful complete computation probability are both met. The long-term average energy consumption, $\psi_{E}\left(\pi \right)$, and the long-term average of successful complete computation probability, $\psi_{S}\left(\pi \right)$, are defined as follows:

(9) $$\begin{array}{c} \psi_{E}\left(\pi \right)=\lim_{T\to \infty }\frac{1}{T}\sum_{t=1}^{T}E_{\pi }\left[\nu (S_{t},A_{t})\right], \end{array}$$

(10) $$\begin{array}{c} \psi_{S}\left(\pi \right)=\lim_{T\to \infty }\frac{1}{T}\sum_{t=1}^{T}E_{\pi }\left[c\left(S_{t},A_{t}\right)\right], \end{array}$$

where $S_{t}\in S$ and $A_{t}\in \left\{0,1,2\right\}$ are the global state and action at time *t*, respectively. From (9) and (10), the constrained stochastic game can be formulated as follows:

(11) $$\begin{array}{cc} \mathrm{Minimize}: & \psi_{E}\left(\pi \right)\\ \mathrm{Subject}\;\mathrm{to}: & \psi_{S}\left(\pi \right)\geq \gamma_{S}, \end{array}$$

where $\gamma_{S}$ is the target average successful complete computation probability. The constrained Nash equilibrium is the solution of the formulated constrained stochastic game. Let $\pi^{*}$ be the best response policy; it is the constrained Nash equilibrium when the following condition is satisfied

(12) $$\begin{array}{c} \psi_{E}\left(\left(\pi^{i*},\pi^{-i*}\right)\right)\leq \psi_{E}\left(\left(\pi^{i},\pi^{-i}\right)\right), \end{array}$$

where $\pi^{i}$ and $\pi^{-i}$, respectively, denote a stationary policy of UAV *i* and a stationary policy of all UAVs excluding UAV *i*, and $\pi^{*}=\left(\pi^{i*},\pi^{-i*}\right)$. We use a linear programming (LP) approach to obtain $\pi^{*}$. Let $\varphi^{i,\pi^{-i}}\left(S^{i},A^{i}\right)$ be the stationary probability in local state $S^{i}$ and action $A^{i}$ of UAV *i* given the policies of the other UAVs $\pi^{-i}$. The LP problem corresponding to (11) can be expressed as follows:

(13) $$\begin{array}{cc} \; & \min_{\varphi (S,A)}\sum_{S}\sum_{A}\varphi^{i,\pi^{-i}}\left(S^{i},A^{i}\right)v\left(S^{i},A^{i}\right) \end{array}$$

(14) $$\begin{array}{cc} \; & s.t.\sum_{S}\sum_{A}\varphi^{i,\pi^{-i}}\left(S^{i},A^{i}\right)c\left(S^{i},A^{i}\right)\;\geq \gamma_{S} \end{array}$$

(15) $$\begin{array}{cc} \; & \sum_{A}\varphi^{i,\pi^{-i}}\left(S^{i^{\prime }},A^{i}\right)=\sum_{S}\sum_{A}\varphi^{i,\pi^{-i}}\left(S^{i},A^{i}\right)P^{i}\left[S^{i^{\prime }}|S^{i},A^{i}\right] \end{array}$$

(16) $$\begin{array}{cc} \; & \sum_{S}\sum_{A}\varphi^{i,\pi^{-i}}\left(S^{i^{\prime }},A^{i}\right)=1 \end{array}$$

(17) $$\begin{array}{cc} \; & \varphi^{i,\pi^{-i}}\left(S^{i^{\prime }},A^{i}\right)\geq 0. \end{array}$$

Here, (13) is the objective function to minimize the average energy consumption of UAV *i*, and the constraints are expressed as (14)–(17). (14) implies that the target successful complete computation probability should always be kept higher than the desired target successful complete computation probability $\gamma_{S}$. The constraints in (15), (16), and (17), respectively, represent the Chapman–Kolmogorov equation and the probability properties.

Let $\varphi^{*i,\pi^{-i}}\left(S^{i},A^{i}\right)$ be the optimal solution of the LP problem defined in (13) through (17). The stationary best response policy of UAV *i* can be obtained as follows:

(18) $$\begin{array}{c} \pi^{i*}\left(S^{i},A^{i}\right)=\frac{\varphi^{i*,\pi^{-i}}\left(S^{i},A^{i}\right)}{\sum_{A^{i^{\prime }}}\varphi^{i*,\pi^{-i}}\left(S^{i},A^{i^{\prime }}\right)}. \end{array}$$

The constrained Nash equilibrium ensures that UAV *i* cannot achieve a lower cost by adopting any other stationary policies if the other UAVs do not change their stationary policies. To update the policies of the UAVs, as shown in Algorithm 1, we apply best response dynamics [34]. Note that the interaction among UAVs is expressed through the probability $\lambda^{-i}$. Then, $\lambda^{-i}$ can be obtained by

(19) $$\begin{array}{c} \lambda^{-i}=1-\prod_{i^{\prime }\neq i}1-\lambda^{i^{\prime }}, \end{array}$$

where $\lambda^{i}$ denotes the probability that UAV *i* receives the MGN’s data; it can be expressed as

(20) $$\begin{array}{c} \lambda^{i}=\sum_{S^{i}\neq 0}\varphi^{i,\pi^{-i}}\left(S^{i},A^{i}=1\right). \end{array}$$

**Algorithm 1:** Best response dynamics algorithm.
  Initialize the policies $\pi^{i}$ for ∀i  repeat  for i=1,…, *N* do      Calculate $\lambda^{-i}$ from (19)      Obtain $\pi^{i*}$ by solving the LP problem      Update the multi-policy, π  end for  until $\lambda^{i}$ converge


## 4. Numerical Example

In this section, simulation results are presented to evaluate the energy consumption and complete computation probability of our system. We simulated a UAV-based system with N=4∼8 UAVs, where the UAVs determined whether or not to compute the MGN’s data by using our scheme. We assumed $E_{max}=10$ (the maximum energy capacity of UAVs) and $P_{H}=\left[0.3∼0.7\right]$ (the probability that UAV harvests energy), where [a,b] denotes a random value between *a* and *b*. Furthermore, we also assumed that the initial batteries of all UAVs were fully charged, and the desired target successful complete computation probability was $\gamma_{S}=0.9$. The chosen system parameters are summarized in Table 1. In addition, our system is also compared with the following four schemes:

1. Always: UAVs always receive the MGN’s data.
2. P-based: UAVs receive the MGN’s data with the probability *P*, where *P* is set to 0.7.
3. Rand: UAVs randomly receive the MGN’s data.
4. Con: One UAV always receive the MGN’s data.

**Figure 2:**
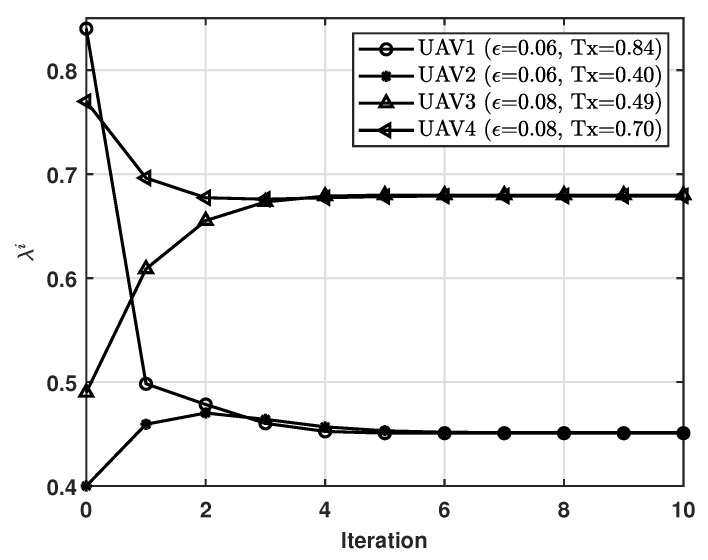
Convergence on the constrained Nash equilibrium policy, where Tx denotes the initial policy of each UAV.

Figure 2 shows the process by which the policies of the UAVs converge on the constrained Nash equilibrium policy.The number of UAVs in the UAV cluster was set to 4, where for $\epsilon =\epsilon_{Tx}=\epsilon_{Rx}$, UAV 1 and UAV 2 each had 0.06, whereas UAV 3 and UAV 4 each had 0.08. As can be seen in this figure, the best response algorithm converged within a few iterations (i.e., six iterations). This result means that our scheme can be implemented in a system without high overhead. We can see that each UAV chose its action by considering the best response relative to the actions of the other UAVs. For example, after the convergence, UAV 1 and UAV 3, which initially had high probabilities of receiving the MGN’s data, had low probabilities; in so doing, their energy consumption was reduced. We can also see that for different values of ϵ, UAVs adaptively chose their action policies, where for ϵ=0.06, $\lambda^{i}=0.45$, whereas for ϵ=0.08, $\lambda^{i}=0.68$.

**Figure 3:**
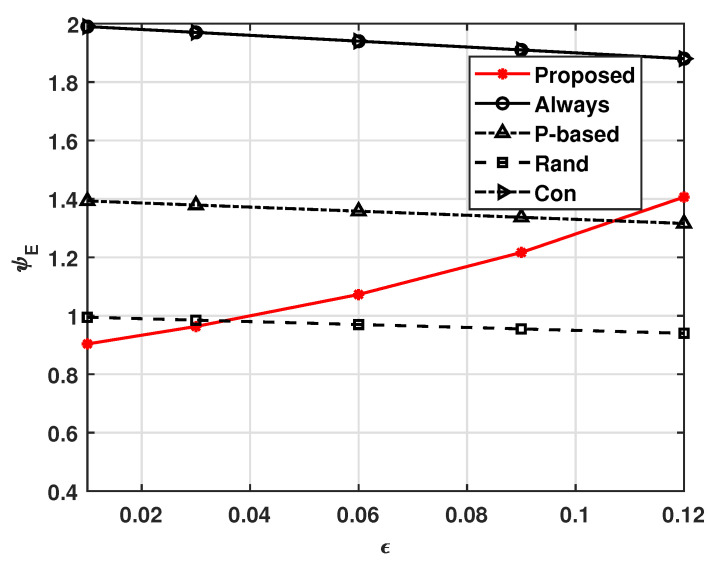
Average energy consumption $\psi_{E}$ versus ϵ.

**Figure 4:**
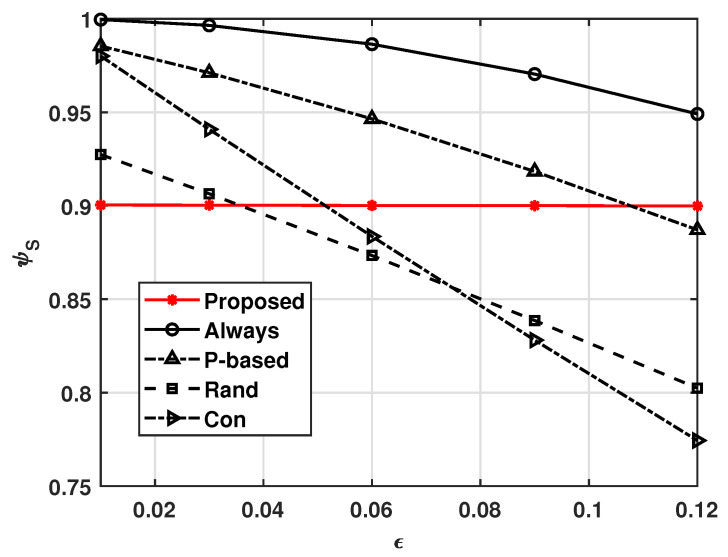
Successful task computational probability $\psi_{S}$ versus ϵ.

Figure 3 and Figure 4, respectively, show the effects of transmission error ϵ on $\psi_{E}$ and $\psi_{S}$, where $\epsilon =\epsilon_{Tx}=\epsilon_{Rx}$. These figures show that our system operates adaptively with varying ϵ. That is, as ϵ increases, more UAVs in our system receive the MGN’s data to avoid situations wherein task data or computed data are not delivered due to ϵ, and the energy consumption is expected to increase as a result. In this way, $\psi_{S}$ can be maintained at $\gamma_{S}$. On the other hand, Figure 3 shows that as ϵ increases, the $\psi_{E}$ values of the other compared schemes decrease slightly; this is because they not only follow a fixed policy, but also do not compute and transmit the data, as a receiving error causes a UAV not to compute a task received from the MGN. Therefore, as shown in Figure 4, the successful complete computation probabilities of the other comparison schemes decrease as ϵ increases. We can also see that the non-cooperation computation scheme, i.e., the Con scheme, suffers from insufficient average successful complete computation probability. This means that cooperative computation schemes have higher average successful complete computation probabilities when the transmission error is high.

**Figure 5:**
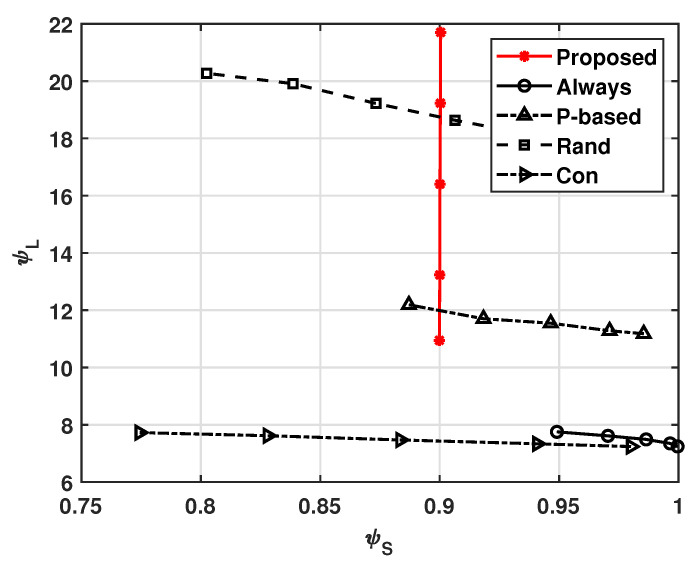
Successful task computational probability $\psi_{S}$ versus cluster lifetime $\psi_{L}$ with various values of ϵ.

Figure 5 shows the cluster lifetime $\psi_{L}$ with respect to $\psi_{S}$, where $\psi_{L}$ is defined as the lifetime of the first dead UAV in the cluster [35] and the initial batteries of all UAVs are fully charged. As shown in vertical red line of Figure 5, our system can prolong $\psi_{L}$ above $\psi_{S}$. That is, as ϵ and $\psi_{L}$ are increased, $\psi_{S}$ is maintained. This is because the UAVs in our system can reduce unnecessary computations by considering the actions of neighbor UAVs. For example, when ϵ=0.01, $\psi_{L}$ = 22; and when ϵ=0.12, $\psi_{L}=11$. For a fixed policy, it should be noted that $\psi_{L}$ cannot be increased without increasing the tolerance of $\psi_{S}$ for the system. We can also see from the result of the Con scheme that the use of cooperative schemes can improve the lifetime of the system.

**Figure 6:**
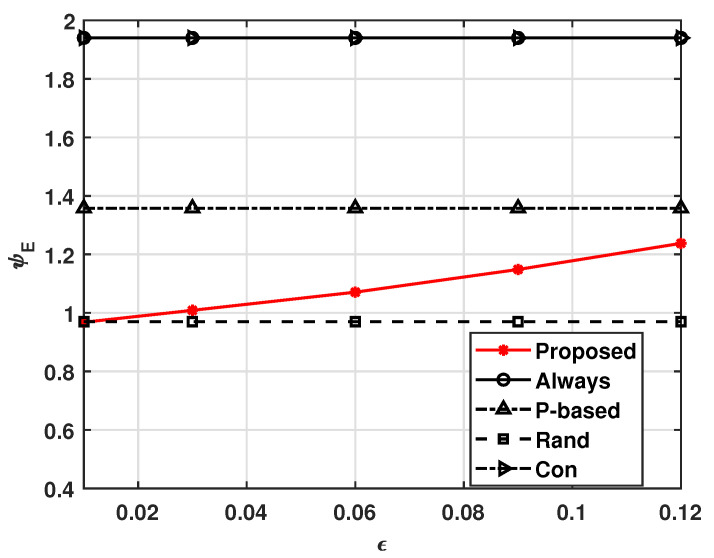
Average energy consumption $\psi_{E}$ versus $\epsilon_{Tx}$, where $\epsilon_{Rx}=0.06$.

**Figure 7:**
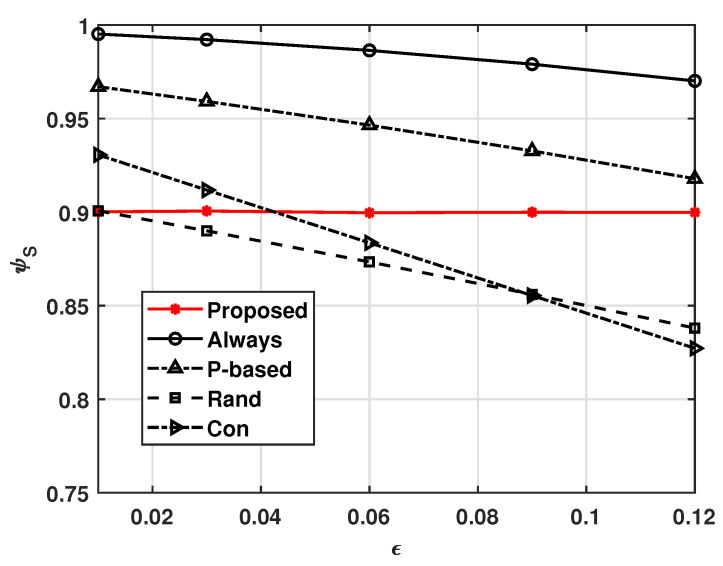
Average energy consumption $\psi_{S}$ versus $\epsilon_{Tx}$, where $\epsilon_{Rx}=0.06$.

Figure 6 and Figure 7 show the effects of uneven transmission error, i.e., $\epsilon_{Tx}\neq \epsilon_{Rx}$, on $\psi_{E}$ and $\psi_{S}$, respectively, where $\epsilon_{Rx}=0.06$ and $0.01\leq \epsilon_{Tx}\leq 0.12$. From these results, we can see that our scheme operates adaptively when $\epsilon_{Tx}$ is changed, as shown in previous results. That is, as $\epsilon_{Tx}$ increases, $\psi_{E}$ is increased, and $\psi_{S}$ is maintained. We can also see that the $\psi_{E}$ values of other schemes remain constant regardless of $\epsilon_{Tx}$, because those policies are fixed. Then, the successful complete computation probabilities of other schemes decrease as $\epsilon_{Tx}$ increase.

**Figure 8:**
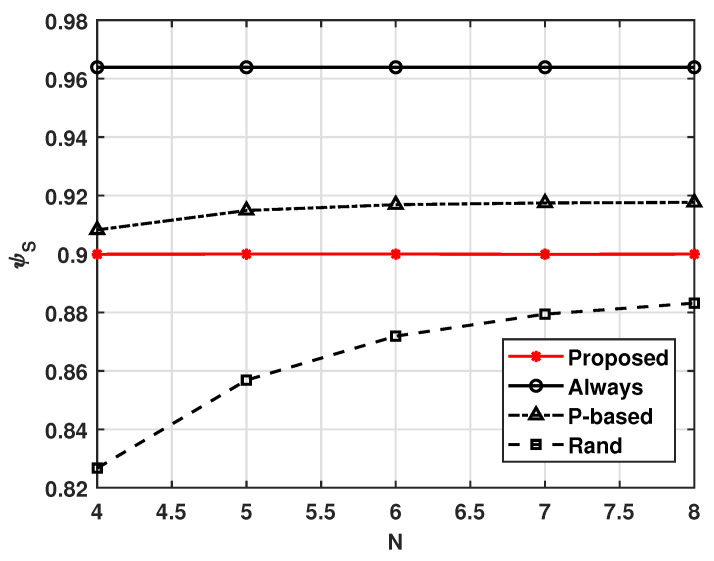
Average energy consumption $\psi_{S}$ versus *N*.

**Figure 9:**
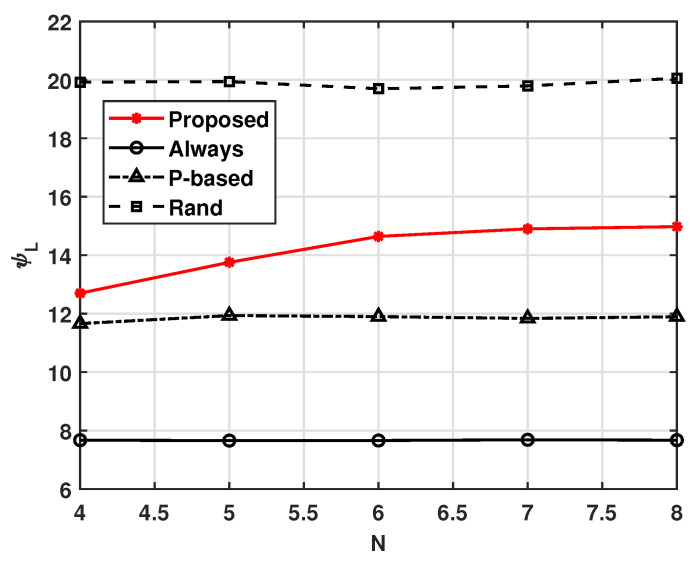
Cluster lifetime $\psi_{L}$ versus *N*.

Figure 8 and Figure 9 show the effect of the number of UAVs in the cluster, *N*. From these figures, we can see that as *N* increases, the cluster lifetime $\psi_{L}$ decreases. This is because the probability that at least one UAV receives the MGN’s data increases with increasing *N*. In this situation, the average successful complete computation probability $\psi_{S}$ can be maintained, as shown in Figure 8.

## 5. Conclusions

In this paper, we propose a UAV-based edge computing system for MGNs which leverages spatial correlation to reduce unnecessary computation and transmission of the MGN’s data. In this system, geographically proximate UAVs decide whether or not to accept a computation request from the MGN by considering the actions of other UAVs. To minimize the number of acceptances and the total amount of computing power used while maintaining the desired successful complete computation probability, a stochastic game model with constraints is formulated, and a multi-policy is obtained by a best response algorithm. The results show that our system can reduce unnecessary energy consumption compared to the probabilistic acceptance scheme and achieve a longer network lifetime, $\psi_{L}$ while maintaining the successful complete computation probability, $\psi_{S}$, above a desired level, $\gamma_{S}$. For example, for $\gamma_{S}=0.9$, $\psi_{S}$ can be maintained at 0.9; for ϵ=0.06, $\psi_{L}=22$; and for ϵ=0.12, $\psi_{L}=11$, respectively. In addition, these findings show that our system operates adaptively even when the operating environment (e.g., the number of UAVs in the cluster and transmission failure) changes. In this paper, we focused on the energy consumption and complete computation probabilities of multiple UAVs. Thus, as future work, for a more accurate performance evaluation from the processed data perspective, we need to see the accuracy for an average data processed by the FedAvg algorithm. Furthermore, we also need to see the energy consumption for the UAV system using retransmission technique.

## Figures and Tables

**Table 1:** System parameters.

Parameter	$E_{\max}$	$P_{H}$	$\gamma_{S}$	*N*
Value	10	[0.3, 0.7]	0.9	4∼8

## Data Availability

Not applicable.
